# Specialities

**Published:** 1859-02

**Authors:** 


### *Specialities.*

Of late it has become very common for medical men to "devote special attention," to the treatment of some particular class of diseases, and although the custom has been greatly opposed by the profession at large, it has gradually gained ground until, at the present time in our larger cities, we find special practitioners for the diseases of almost every organ in the human body. We have not only Oculists and Aurists but Lithotomists, Chiropodists and Orthopedists. Before the profession was so much crowded as it is now, all medical men were supposed to be equally skilled in every branch of medicine, surgery and dentistry, so that after extracting a few teeth in the morning, visiting a case of fever, or amputating a leg at noon, the practitioner often closed his labors by attending a woman in labor, at night.

Skill in Medicine and Surgery can only be acquired by practice, and however perfect one's knowledge of these arts may be in theory, he is scarcely more competent for the practical pursuit than the merest novice, unless he has gained dexterity from extensive and long continued practice. In every community the more common diseases and accidents are best understood by the general practitioner, and while he would diagnosticate and treat a case of fever, pneumonia or dysentery, correctly and with skill, he might perhaps be at a loss to pronounce an opinion on, much more to treat an obscure case of vesical calculus, ovarian tumor, incipient phthisis or acute glaucoma. The first time one undertakes the physical exploration of the chest for the purpose of detecting disease of the heart or lungs, he finds great difficulty in distinguishing the various sounds that meet his ear—often he can hear none at all—however good an examination he might be qualified to pass on the murmurs, ronchi and tones which are found in the different morbid conditions of the thoracic viscera. In time however, his ear, educated by practice, readily detects the breezy murmur of healthy respiration, the coarse rhonchus of bronchitis, the fine crepitations of pneumonia, the blowing sound of valvular disease of the heart, and all the modifications of sound which indicate Pulmonic or Cardiac disease. It is so difficult to acquire skill in Ascultation and percussion, that nothing is more common, than to hear physicians say they regard the information which these afford in the diagnosis of disease as of very little value, and many practitioners seldom, if ever attempt to employ them. Is it not reasonable to suppose that a person who has made the diagnosis and treatment of disease of the chest his especial study should possess more skill than general practitioners ordinarily do? The readiness with which physicians recommend patients laboring under obscure pulmonary complaints to consult Dr. Bowditch of Boston is a practical answer in the affirmative.

It is a notorious fact that no class of diseases is so badly treated by medical men generally, as those of the eye and ear. This is attributable to several causes. The comparatively small number of cases of ocular disease which each practitioner ordinarily meets, and the consequent difficulty of distinguishing them, offer insuperable obstacles to the acquisition of more than a very small amount of skill in their treatment, while the delicate manipulations required for the performance of even the simplest operations on the eye, demand daily and continual practice to ensure any degree of skill whatever.

We occasionally meet individuals whose sight has been irremediably lost in consequenee of the unskillful attempt of some medical man to perform one of the most simple operations in opthalmic surgery.

These facts certainly afford a strong argument in favor of the practice of medical and surgical specialties. On the other hand the disposition to overlook all other diseases, and to attribute every pathological condition to derangement of those organs which the specialist has made his particular study to treat, that we sometimes find, seems to present a very potent objection to the subdivision of medical and surgical practice into specialties. This objection however, is rather specious than real, since it applies only to those who are merely pretenders to skill or to a particular class of diseases which has not as yet been thoroughly investigated and consequently is not well understood. —*Maine Medical & Surgical Reporter.*

